# Perceived stress and symptoms of post-traumatic stress disorder in nurses: A moderated mediation model of maladaptive cognitive emotional regulation and psychological capital

**DOI:** 10.3389/fpsyt.2022.902558

**Published:** 2022-11-23

**Authors:** Mengxin Xue, Yuan Yuan, Hong Chen, Yongbing Liu, Minghui Dai, Huiping Sun, Jiling Qu, Ting Zhou, Jingxin Zhou, Junchao Qu, Yuan Bu, Siqi Ji, Yicheng Hu, Zhenshuai Yao, Yanbing Feng, Xinyi Gu

**Affiliations:** ^1^School of Nursing and Public Health, Yangzhou University, Yangzhou, China; ^2^Affiliated Hospital of Yangzhou University, Yangzhou, China; ^3^People's Hospital of Northern Jiangsu Province, Yangzhou, China

**Keywords:** nurses, symptoms of post-traumatic stress disorder, perceived stress, cognitive emotional regulation, psychological capital

## Abstract

Nurses often face a variety of work-related and life-related stresses that make them more prone to symptoms of post-traumatic stress disorder (PTSD), yet the underlying mechanism of this association is poorly understood. To address this research gap, we investigated the mediating role of maladaptive cognitive emotion regulation strategies in the relationship between perceived stress and PTSD symptoms, and explored whether psychological capital could moderate the direct or indirect effects between perceived stress and PTSD symptoms. Nurses (*N* = 723) completed a questionnaire about perceived stress, PTSD symptoms, maladaptive cognitive emotion regulation strategies and psychological capital. After controlling for gender, age and work department, perceived stress was positively correlated with PTSD symptoms. Maladaptive cognitive emotion regulation strategies partially mediated this relationship. Psychological capital moderates the effects of perceived stress and maladaptive cognitive emotion regulation strategies on PTSD symptoms. Specifically, the positive correlation between perceived stress and PTSD symptoms was stronger among nurses with low levels of psychological capital than among nurses with high levels of psychological capital. At the same time, the positive correlation between maladaptive cognitive emotion regulation strategies and PTSD symptoms was stronger in nurses with a low level of psychological capital. Therefore, cognitive strategies and interventions oriented toward psychological capital may alleviate the PTSD symptoms of nurses in stressful situations.

## Introduction

Post-traumatic stress disorder (PTSD) is a long-term psychosomatic disorder with delayed onset that develops in individuals following a threat or catastrophic traumatic event. According to the Diagnostic and Statistical Manual of Mental Disorders (DSM-5), clinical symptoms include repeated recurrence of traumatic experiences, continued avoidance of stimuli associated with traumatic events, negative changes in cognition and mood, and continued increased alertness (1). Worldwide, the prevalence of PTSD is reported to be 3.9% in the general population and 5.6% in those with traumatic exposure (2). However, nurses are at high risk for developing PTSD symptoms. The chronic job stressors and exposure to traumatic events are risk factors for PTSD symptoms in nurses (3). Nurses have both direct and indirect exposure to trauma, and their work may be a source of additional pressures such as workplace issues (violent behavior, high workload, patient suicide) and relationship problems (conflict with colleagues, workplace bullying, lack of support), etc. (4). In a recent integrative review, 6.7–95.7% of nurses showed at least **one** symptom of PTSD, and 8.5–20.8% met the criteria for PTSD (4). In addition, nurses have often been on the front lines during the COVID-19 pandemic, taking on more stress and work. According to one survey, 74.4% of nurses experienced moderate to severe perceived stress. 17.1% of nurses may have been diagnosed with PTSD (5). Life events are also important factors affecting the level of PTSD in nurses (6). A growing number of studies have shown that PTSD in nurses not only affects their physical and mental health, but also leads to adverse outcomes such as empathy fatigue, job burnout, low job satisfaction and poor quality of care (7, 8). Therefore, it is of great significance to pay attention to the factors influencing PTSD symptoms in nurses and to understand the relevant mechanisms underlying these symptoms in order to maintain and even improve nurses' physical and mental health.

It has been proved that the occurrence of disastrous and traumatic events will cause mental disorders (9), and the influence of objective events is determined to some extent by an individual's perception of the pressure resulting from the event (10). Compared with objective measurement of stressors, the perception of a stressful environment influences the individual's response more strongly (11). Lazarus and Folkman define perceived stress as an individual's response to an environment perceived as a threat to their ability and health (12). Surveys of perceived stress levels and PTSD symptoms in different populations have reportedly found a positive correlation between the severity of PTSD symptoms and perceived stress (13). A survey of medical workers in intensive care units found that respondents with moderate to high perceived stress scores were more likely to be diagnosed with PTSD than those with low perceived stress scores (5). That is, perceived stress may increase the severity of PTSD symptoms (14). Although increasing numbers of studies have confirmed the effect of perceived stress on PTSD, little is known about the role of negative cognition and psychological capital in this relationship. In order to provide better prevention and intervention strategies and reduce the risk of PTSD in nurses, this study explored a moderated mediation model to reveal the possible mechanism behind this relationship.

According to Garnefski et al., the general concept of emotion regulation can be regarded as a cognitive style of managing the intake of emotionally arousing information, which encompasses a broad range of cognitive, behavioral, emotional, and physiological responses (15). Garnefski et al. conceptualized cognitive emotional regulation as adaptive cognitive emotional regulation strategies (adaptive CERS) (e.g., positive reappraisal, positive focusing, putting into perspective, refocusing on planning and acceptance) and maladaptive cognitive emotional regulation strategies (maladaptive CERS) (e.g., self-blame, rumination, other-blame and catastrophizing) (15). The use of cognitive styles of maladaptive CERS has been reported to increase an individual's tendency to experience emotional problems, often associated with mental disorders (e.g., depression, anxiety, PTSD). Conversely, the use of adaptive CERS may prevent the onset and persistence of mental illness, often associated with better indicators of mental health (e.g., resilience) (16–19). The application of different strategies for cognitive emotion regulation when a person is faced with traumatic events or stress may lead to different mental health outcomes (20). Studies have confirmed that perceived stress is positively correlated with maladaptive CERS (21). Maladaptive CERS mediate the relationship between traumatic events or stress and mental health outcomes (16). According to theoretical studies and the existing literature, maladaptive CERS may mediate the relationship between perceived stress and PTSD symptoms. Therefore, in this study, we proposed the **first** hypothesis:

Hypothesis 1: Maladaptive cognitive emotion mediates the relationship between perceived stress and PTSD symptoms.

Although stress and Maladaptive cognitive emotion might influence PTSD symptoms, not all nurses with higher levels of stress and Maladaptive cognitive emotion experience more PTSD symptoms. Hence, it is vital to investigate the influential factor that might moderate the associations of stress and Maladaptive cognitive emotion with PTSD among nurses. Although stress has an adverse effect on PTSD symptoms (22), the diathesis–stress model suggests that people with vulnerable characteristics in stressful situations will have an increased likelihood of developing mental disorders (23). In other words, having good personality traits can modulate the psychological response to stress. Our research focuses on the characteristics of psychological capital, which Luthans et al. define as “the positive psychological state of individuals in the process of growth and development,” including **four** core components: self-efficacy, hope, optimism and resiliency (24). A large number of studies have shown that psychological capital is an important protective factor for individual mental health and helps to resist the adverse effects of stress (such as psychological distress and job burnout) (25, 26). It also has a significant positive impact on satisfaction, organizational commitment and workplace wellbeing, and is a positive resource for combating employee stress symptoms and staff turnover (27). Psychological capital has been reported to be negatively correlated with negative coping, and can indirectly affect a person's psychological distress level through their coping style (26). Psychological capital is negatively associated with maladaptive CERS, and this negative cognitive emotional coping may significantly impede the development of psychological capital (19). To our knowledge, although psychological capital acts as an important protective factor against the onset and persistence of psychological distress, the associations among psychological capital modulating perceived stress, maladaptive CERS, and PTSD symptoms have not been established. Therefore, in order to reduce the incidence of mental disorders among nurses, it is necessary to study the protective mechanism of psychological capital. Once it is understood how psychological capital moderates the association between perceived stress and maladaptive CERS/PTSD symptoms, nursing managers can design effective intervention plans to reduce the occurrence of PTSD symptoms in nurses. Therefore, we propose the following hypotheses:

Hypothesis 2: Psychological capital moderates the relationship between perceived stress and PTSD symptoms.

Hypothesis 3: Psychological capital moderates the relationship between perceived stress and maladaptive CERS.

In addition, psychological capital can help individuals to alleviate the adverse effects of maladaptive CERS on mental health. Studies have shown that a high level of psychological capital has a protective effect on psychological vulnerability, and can buffer the adverse effects of maladaptive CERS on psychological vulnerability (28). However, whether psychological capital moderates the relationship between maladaptive CERS and PTSD symptoms needs further verification. Therefore, in this study, we propose the fourth hypothesis:

Hypothesis 4: Psychological capital moderates the relationship between maladaptive CERS and PTSD symptoms.

**Figure 1:**
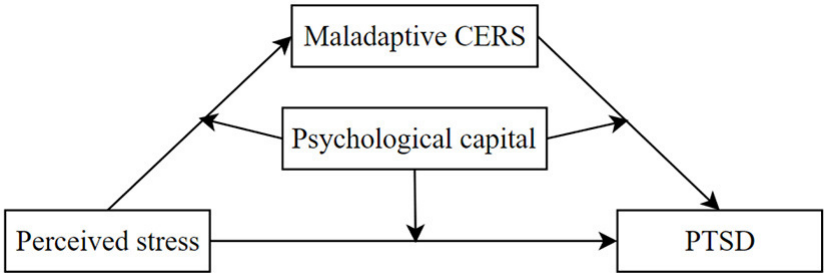
Hypothesis model.

To date, no study has investigated the regulatory and mediating mechanisms among perceived stress, maladaptive CERS, PTSD symptoms, and psychological capital. Therefore, based on theories and the existing literature, we constructed a moderated mediation model (Figure 1) to investigate the mediating and regulating mechanisms of perceived stress in predicting PTSD symptoms, so as to provide empirical support and theoretical guidance for the occurrence and persistence of PTSD symptoms among nurses.

## Materials and methods

### Participants and procedure

Convenience sampling was used in this cross-sectional study, which was conducted in eight hospitals in Jiangsu Province from September 12 to September 22, 2021. Data were collected through an online survey platform (https://www.wjx.cn/), and all information was obtained in the form of an anonymous self-report. Candidates eligible to participate in this study were actively working registered nurses, without a history of psychiatric disorders, aged 18 years or older, and with at least 1 year of experience, who also volunteered to participate in this study. Intern nurses, refresher nurses, retired nurses, nurses working in non-clinical posts, and nurses who experienced life events were not allowed to participate in this study. The sample size was calculated using the formula N = ${\text{Z}}_{\text{α}/2}^{2}\pi (1-\pi$)(DEFF)/ d^2^ (π= Estimate of the expected proportion, d = Desired level of absolute precision, DEFF = Estimated design effect) (29). In this study, α= 0.05, Z_α/2_= 1.96, π= 17.1%, DEFF = 1, When the value of π is outside 20~80%, d = 3%, Considering 10% dropout rates, we calculated that the total sample size was 667 in this study (5). A total of 1,424 people participated in the survey, 83 questionnaires that did not fit the nursing profession were excluded, as were 187 questionnaires that took < 500 s to fill. Four hundred thirty-one registered nurses were excluded because they had experienced at least one life event in the list of life events such as divorce, death of a spouse, heavy debt, robbery, moving, serious illness or serious injury to a family member, and death of a family member. A total of 723 data pieces were available for analysis. Data collection for this study was approved by the Ethics Committee of the School of Nursing, Yangzhou University (No: YZUHL2021028). During the study investigation, participants were told they could withdraw from the study at any time.

### Measures

#### The PTSD checklist for DSM-5 (PCL-5)

The Post-traumatic Stress Disorder Screening form is based on the Diagnostic and Statistical Manual of Mental Disorders, Edition 5 (DSM-5) criteria (30). The questionnaire consists of 20 items, including intrusive symptoms (criterion B: questions 1–5), avoidance symptoms (criterion C: questions 6–7), negative alteration in cognition and mood symptoms (criterion D: questions 8–14), and hyperarousal (criterion E: questions 15–20). The questionnaire was rated on a five-point Likert scale of 0 (not at all) to 4 (extremely). The total score is 0–80. A higher score indicates a higher level of PTSD symptoms. The scale has been widely used to measure PTSD symptoms among health care workers (31), with a positive screening cutoff of 33 points. In this study, the Cronbach's alpha was 0.972 for the PCL-5 scale.

#### The Chinese version of the perceived stress scale

The perceived stress scale developed by Cohen et al. was used to measure the self-perceived stress level (32). The scale includes two dimensions, tension and control, with 14 items, each on a five-point Likert scale ranging from 0 (never) to 4 (very common). Scores range from 0 to 56, and the total score is positively correlated with perceived stress. The Chinese version of the scale has been proven to have good reliability and validity (33, 34). In this study, the Cronbach's alpha was 0.691 for the CPSS scale.

#### Maladaptive cognitive emotion regulation strategies (maladaptive CERS)

The Cognitive Emotion Regulation Questionnaire (CERQ) was developed by Garnefski et al., and is used to evaluate strategies for cognitive emotion regulation in the face of negative events (15). The Chinese version of the Cognitive Emotion Regulation Questionnaire (CERQ-C) used in this study has good reliability and validity (35). There were 36 items in the questionnaire, including nine dimensions, five kinds of adaptive CERQ-C (positive reappraisal, positive focusing, putting into perspective, refocusing on planning, and acceptance) and four kinds of maladaptive cognitive emotion regulation strategies (self-blame, rumination, other-blame, and catastrophizing) respectively, and five-point Likert scales from 1 (never) to 5 (always) were used to assign scores. In this study, the CERQ-C maladaptive strategy questionnaire was used and Cronbach's alpha was 0.960.

#### The nurse psychological capital questionnaire (PCQ-R)

This instrument was developed by Luthans et al. to evaluate the psychological capital of employees (36). Luo Hong et al. translated and revised the scale based on the characteristics of nursing work (37), with a total of 20 items, including self-efficacy, hope, resilience and optimism. The questionnaire used a six-point Likert scale, rated from 1 (strongly disagree) to 6 (strongly agree), and the total score was 20–120. The higher the score, the higher the level of psychological capital. This scale has been proved to have good reliability and validity (25, 37). In this study, the Cronbach's alpha was 0.991 for the PCQ-R scale.

#### The patient health questionnaire-9

The Generalized Anxiety Disorder Scale-7 was used to assess the severity of seven depression symptoms in the last 2 weeks (38). The scale uses a 4-point Likert scale from 0 (not at all) to 3 (nearly every day), with an overall score range of 0 to 27. The scores of 5, 10 and 20 were the threshold of mild, moderate and severe depression, respectively (38). In this study, the Cronbach's alpha of GAD-7 was 0.954.

### Statistical analyses

The data were analyzed using IBM SPSS Version 26, and the SPSS macro PROCESS was used to test the moderated mediation model (39). The analysis was carried out in four steps. First, the basic characteristics of nurses were analyzed by descriptive statistics. The Mann-Whitney U nonparametric test and Kruskal-Wallis nonparametric test were used to compare the PTSD scores of nurses with different demographic characteristics. Second, the Harman single-factor test was used for a common method deviation test (40). Spearman correlation was used for analysis of correlation and the basic situation of measured variables was described. Third, Model 4 (a simple mediation model) in the SPSS macro PROCESS was used to analyze the mediating effect of maladaptive cognitive emotion regulation strategies on the relationship between perceived stress and PTSD symptoms. Fourth, the SPSS macro PROCESS model 59 was used to analyze the moderating effect of psychological capital on the direct or indirect relationship between perceived stress and PTSD symptoms. All study variables except covariables were standardized before PROCESS analysis. The bootstrapping method (5,000 bootstrapping samples) with 95% confidence intervals (CIs) was conducted to detect the significance of the effects (39).

## Results

### Characteristics of the research participants

The 723 nurses were aged from 22 to 59 years, with an average of 32.91 (±6.73) years, and their working years ranged from 1 to 40, with an average of 11.34 (±7.55). The demographic characteristics of the study participants and univariate analysis for the factors related to the level of PTSD symptoms is shown in Table 1. The results showed that gender (*p* = 0.018), age (*p* = 0.016), work department (*p* = 0.036), and depression (*p* < 0.001) were associated with PTSD symptoms.

**Table 1:** Demographic characteristics of the study participants and univariate analysis for the factors related to the level of PTSD symptoms.

**Variable**	***n* (%)**	**PTSD** **Median (interquartile range)**	**Z/H**	***p***
Age			−2.415^a^	0.016
< 30	229 (31.7)	8.00 (1.00, 20.00)		
≥30	494 (68.3)	12.00 (3.00, 20.00)		
Sex			−2.369^a^	0.018
Male	12 (1.7)	1.50 (0.00, 14.75)		
Female	711 (98.3)	12.00 (2.00, 20.00)		
Marital status			−1.529^a^	0.126
Single	150 (20.7)	8.00 (1.00, 20.00)		
Married	573 (79.3)	12.00 (2.00, 20.00)		
Working years			3.931^b^	0.140
≤ 5	145 (20.1)	8.00 (0.00, 20.50)		
6-10	261 (36.1)	13.00 (2.00, 20.00)		
≥11	317 (43.8)	12.00 (2.50, 20.00)		
Education degree			3.211^b^	0.201
Below bachelor's degree	54 (7.5)	8.50 (1.00, 20.00)		
Bachelor's degree	664 (91.8)	12.00 (2.00, 20.00)		
Above bachelor's degree	5 (0.7)	3.00 (0.00, 14.00)		
Work department			10.302^b^	0.036
Internal medicine	294 (40.7)	9.00 (1.00, 20.00)		
Surgical	225 (31.1)	12.00 (1.00, 20.00)		
ICU	82 (11.3)	14.50 (1.00, 26.00)		
Emergency department	57 (7.9)	15.00 (4.00, 24.00)		
Other departments	65 (9.0)	15.00 (3.00, 23.50)		
Depression			248.593^b^	< 0.001
None	313 (43.3)	3.00 (0.00, 10.00)		
Mild	298 (41.2)	17.00 (8.00, 21.00)		
Moderate	98 (13.6)	32.50 (20.00, 40.25)		
Severe	14 (1.9)	41.50 (12.25, 64.75)		

a Mann-Whitney U nonparametric test.

b Kruskal-Wallis nonparametric test.

### Common method deviation test

The Harman single factor test was used to test common method deviation. The results showed that there were seven factors whose initial eigenvalue was >1. The first factor explained 37.46% of the total variation, which was less than the critical value of 40%, indicating that there was no serious common method bias.

### Correlation analysis of measurement variables

For preliminary analysis of the relationships between variables, descriptive statistics and correlation analysis were conducted, as shown in Table 2. The results showed that maladaptive CERS (*r* = 0.182, *p* < 0.001) and PTSD symptoms (*r* = 0.365, *p* < 0.001) were positively correlated with perceived stress. In addition, perceived stress (*r* = −0.389, *p* < 0.001), maladaptive CERS (*r* = −0.326, *p* < 0.001) and PTSD symptoms (*r* = −0.429, *p* < 0.001) were negatively correlated with psychological capital. Finally, maladaptive CERS was positively correlated with PTSD symptoms (*r* = 0.674, *p* < 0.001).

**Table 2:** Descriptive statistics and correlations for all variables.

**Variable**	**M**	**SD**	**1**	**2**	**3**	**4**
Perceived stress	23.93	6.92	1.00			
Maladaptive CERS	36.39	11.68	0.182^***^	1.00		
PTSD	14.19	13.95	0.365^***^	0.674 ^***^	1.00	
Psychological capital	88.47	21.03	−0.389^***^	−0.326^***^	−0.429^***^	1.00

*N* = 723;

*** *p* < 0.001.

### Testing for mediation effect

**Figure 2:**
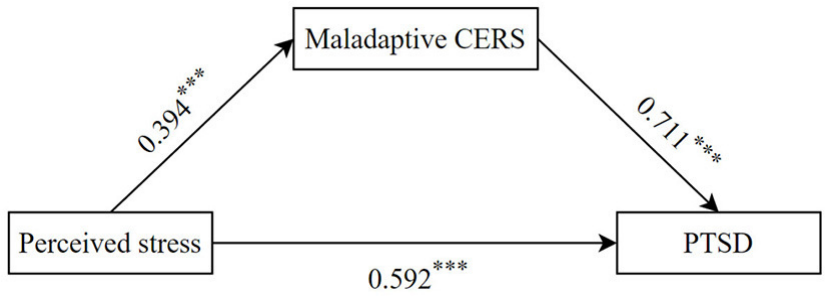
The mediating effect in the association between perceived stress and PTSD symptoms. ***p < 0.001.

According to hypothesis 1, we evaluated whether maladaptive CERS plays a mediating role between perceived stress and PTSD symptoms, as shown in Figure 2. Model 4 (a simple mediation model) in the SPSS macro PROCESS was used to test the mediation effect. After controlling for age, gender and work department, perceived stress had a significant predictive effect on PTSD symptoms *(*β = 0.592, *p* < 0.001). Perceived stress significantly predicted maladaptive CERS (β = 0.394, *p* < 0.001), and maladaptive CERS significantly predicted PTSD symptoms *(*β = 0.711, *p* < 0.001). Bootstrapping analysis further showed that the indirect effect of maladaptive CERS was significant (indirect effect = 0.280, SE (Standard Error) = 0.044, 95%CI = [0.198, 0.368]), and the direct effect of perceived stress and PTSD symptoms was significant (β = 0.592, *p* < 0.001). Maladaptive CERS partially mediated the relationship between perceived stress and PTSD symptoms, accounting for 32.11% of the total effect. Therefore, hypothesis 1 is supported.

### Testing for moderated mediation

**Figure 3:**
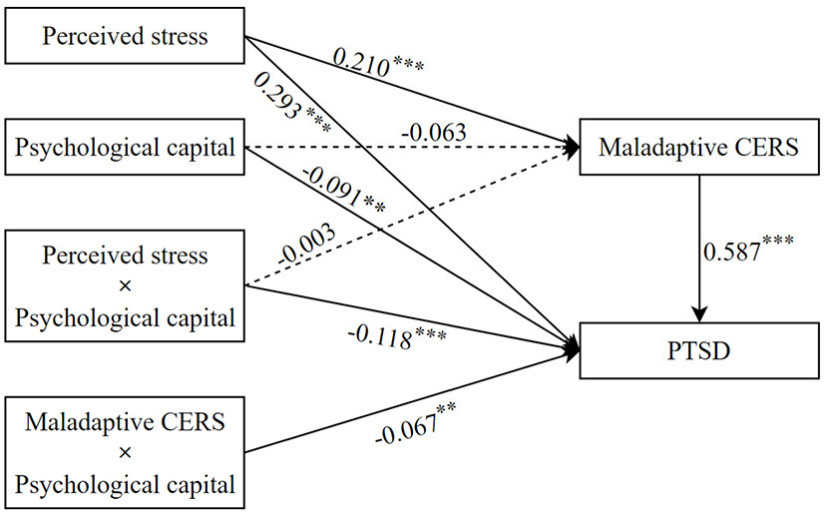
The moderating effect of psychological capital on the relationship between perceived stress and PTSD symptoms. **p < 0.01; ***p < 0.001.

**Figure 4:**
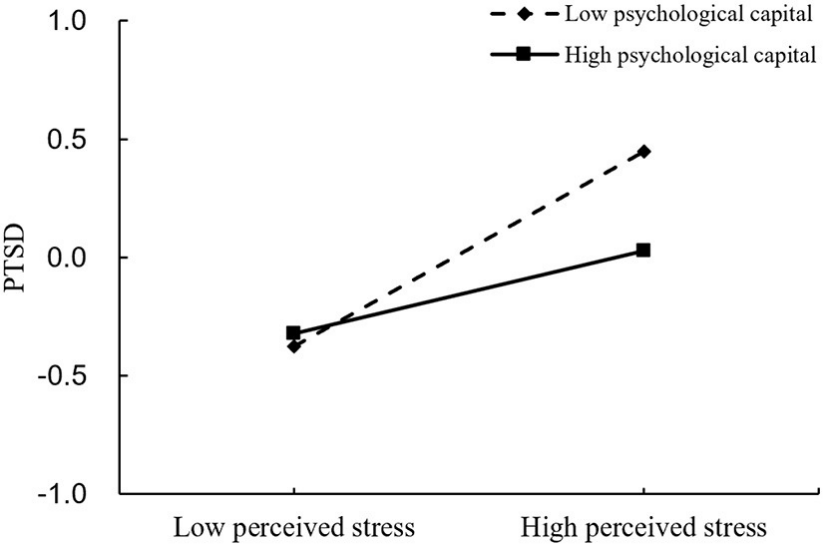
Association between perceived stress and PTSD symptoms at higher and lower levels of psychological capital.

According to hypothesis 2, we tested whether psychological capital played a moderating role between perceived stress and PTSD symptoms, as shown in Figure 3. After controlling for age, gender, and work department, perceived stress was positively associated with PTSD symptoms (β = 0.293, *p* < 0.001), whereas psychological capital was negatively associated with PTSD symptoms (β = −0.091, *p* < 0.01). Furthermore, psychological capital significantly moderated the direct association between perceived stress and PTSD symptoms (β = −0.118, *p* < 0.001). To further examine the effect of different levels of psychological capital on PTSD symptoms, we divided psychological capital into a low level psychological capital group (1 standard deviation below the mean) and a high level psychological capital group (1 standard deviation above the mean) for simple slope analysis. As shown in Figure 4, the effect of perceived stress on PTSD symptoms has a steeper slope for people with low levels of psychological capital than for people with high levels of psychological capital. For those with low levels of psychological capital, higher perceived stress was associated with higher PTSD symptoms (β = 0.411, *p* < 0.001), whereas for those with high levels of psychological capital, this association was weakened (β = 0.175, *p* < 0.001). This implies that psychological capital inhibits the positive relationship between perceived stress and PTSD symptoms. Therefore, hypothesis 2 is supported.

**Figure 5:**
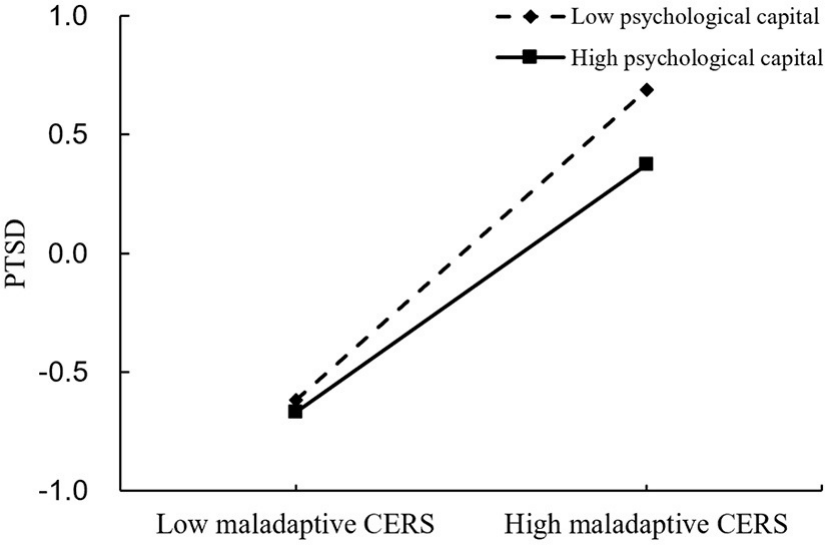
Association between maladaptive CERS and PTSD symptoms at higher and lower levels of psychological capital.

We examined the potential moderating role of psychological capital in the indirect associations between perceived stress and PTSD symptoms *via* maladaptive CERS, as shown in Figure 3. The results showed that the interaction between maladaptive CERS and psychological capital was significant in predicting PTSD symptoms (β = −0.067, *p* < 0.01); however, the interaction between perceived stress and psychological capital was not significant in predicting maladaptive CERS (β = −0.003, *p* > 0.05). Thus, hypothesis 3 is not supported. A simple slope analysis is shown in Figure 5, which describes the relationship between maladaptive CERS and PTSD symptoms on two levels of psychological capital (i.e., 1 standard deviation below the mean and 1 standard deviation above the mean). It can be seen that the effects of maladaptive CERS on PTSD symptoms have a steeper slope when compared with individuals with high levels of psychological capital. Higher maladaptive CERS were associated with higher PTSD symptoms in those with low levels of psychological capital (β = 0.654, *p* < 0.001); however, the association was weakened in those with high levels of psychological capital (β = 0.520, *p* < 0.001). This suggests that psychological capital inhibits the positive association between maladaptive CERS and PTSD symptoms, and hypothesis 4 is thereby supported.

## Discussion

Although there is growing evidence that stress has a significant effect on PTSD symptoms, few studies have explored the role of maladaptive CERS and psychological capital in this relationship using a process-oriented approach. Based on previous studies and theoretical frameworks, we constructed a moderated mediation model. In this study, we found that the relationship between perceived stress and PTSD symptoms is partly mediated by maladaptive CERS and moderated by psychological capital. These findings may help in the development of targeted interventions aimed at reducing nurses' risk of developing PTSD symptoms in stressful situations.

Our results suggest that maladaptive CERS mediate the association between perceived stress and PTSD symptoms, and that higher levels of perceived stress are associated with higher levels of maladaptive CERS, which in turn are associated with higher levels of PTSD symptoms. In addition, our findings showed that maladaptive CERS, perceived stress and PTSD symptoms were positively correlated, which confirms the findings of previous studies (18, 21, 22). Our results are consistent with previous findings that the use of maladaptive CERS is an important mechanism underlying the negative effects of stress on psychological dysfunction (41). When nurses use maladaptive CERS more frequently, PTSD symptoms increase, with the increase of perceived stress. This suggests that maladaptive CERS are important risk factors for PTSD symptoms in nurses. Nurses should avoid maladaptive cognitive emotional regulation strategies in order to prevent the adverse effects of perceived stress on PTSD symptoms. In future research, further investigation is needed to determine which specific cognitive emotion regulation strategies are more protective or confer more risk for PTSD symptoms. In conclusion, maladaptive CERS induced by perceived stress may be an important risk factor for the development of PTSD symptoms.

In addition, psychological capital was negatively correlated with perceived stress and PTSD symptoms in our study, which is consistent with previous findings (42, 43). Wang et al. showed that perceived stress was more likely to lead to negative emotions among medical students with lower levels of psychological capital (42). Our results support our hypothesis that psychological capital is a mediator of the direct relationship between perceived stress and PTSD symptoms and, more specifically, perceived stress is an important risk factor for developing PTSD symptoms in nurses with low levels of psychological capital. Hobfoll proposed the conservation of resources theory, which argues that valuable resources play a positive role in the individual stress response. These resources include material resources, power, interpersonal relationships and positive psychological factors (44, 45). This means that psychological capital, as a positive psychological quality, is regarded as a protective factor for mental health, so that individuals can still have good adaptability in the face of environmental pressure. When facing the pressures of life and work, individuals with a higher level of psychological capital can mobilize positive psychological resources and alleviate the negative impact of pressure with an optimistic attitude, strong self-efficacy and resilience.

The moderating effect model of psychological capital holds that psychological capital influences individual, group and organizational outcome variables through its moderating effect, which is supported by empirical research (46). As hypothesized, psychological capital moderated the association between maladaptive CERS and PTSD symptoms and, in particular, the adverse effects of maladaptive CERS on PTSD symptoms were greater for nurses with lower levels of psychological capital than for nurses with higher levels. Adaptive cognitive emotion regulation and psychological capital have been reported to have a role in regulating and maintaining mental health (28). Good psychological capital, with positive psychological qualities of self-efficacy, hope, resilience and optimism, helps an individual to deal with negative events with a positive attitude, promotes the stimulation of individual positive emotions, and alleviates the negative effects of maladjustment on PTSD symptoms.

In conclusion, high psychological capital moderated the effects of perceived stress on PTSD symptoms, allowing individuals to cope effectively with stress and alleviating the effects of maladaptive CERS on PTSD symptoms. This means that improving nurses' level of mental capital is an important measure to prevent mental health problems. However, contrary to our hypothesis, psychological capital does not play a moderating role in perceived stress and maladaptive CERS; that is, psychological capital does not protect against the negative effects of perceived stress on maladaptive CERS. A possible explanation is that maladaptive CERS are often associated with mental health problems, such as depression and PTSD symptoms (16, 18). However, adaptive CERS are usually associated with positive psychological factors, such as resilience and psychological capital (17, 19). Given the lack of research, larger studies are needed to confirm these findings.

Our study provides new evidence of an association between the mediating role of maladaptive CERS and the moderating role of psychological capital in the relationship between perceived stress and PTSD symptoms. However, our study has some limitations. First, the cross-sectional design of the study makes it difficult to infer a causal relationship among these variables, and large-scale, multi-center prospective studies are needed to verify the findings. Second, the study excluded registered nurses with traumatic life events, but did not investigate the nurses' traumatic work experience and work stressors, and these traumatic experiences have an important impact on the occurrence and development of PTSD. Third, we observed a very skewed gender distribution in our sample, with 98.3% of the participants being females. The gender-related findings might be totally biased. Further study should be conducted to replicate our findings in a gender-balanced sample. Finally, the data used in this study were all obtained from the supervisor reports of the participants, so the results may be biased, and the interpretation and application of the results should be cautious. Future studies should consider collecting data from multiple sources to improve the objectivity of measurement.

Despite these limitations, there are important practical implications. On the one hand, in our study, maladaptive CERS were a major factor linking perceived stress and PTSD symptoms. It is important to note that cognitive strategy is a modifiable factor, and previous empirical studies have shown that cognitive training can increase the use of adaptive CERS and decrease the use of maladaptive CERS (47, 48). On the other hand, our results suggest that psychological capital plays a moderating role in perceived stress/maladaptive CERS and PTSD symptoms. Studies by Dello et al. show that training courses on psychological capital can significantly improve the level of individual psychological capital, and the effect of the intervention is lasting (49). This means that interventions targeting individual psychological capital may be effective in preventing or alleviating mental health problems. We suggest that health professionals should develop and provide prevention and intervention programs oriented toward cognitive emotion and psychological capital to promote the maintenance of mental health among health workers.

In conclusion, our study constructed a moderated mediation model to examine the relationship between perceived stress and PTSD symptoms. Maladaptive CERS are a partial mediator between perceived stress and PTSD symptoms. In addition, psychological capital moderates the effects of perceived stress and maladaptive CERS on PTSD symptoms. Our findings highlight the importance of evaluating and improving interventions for maladaptive CERS and psychological capital, which may be effective strategies to prevent or reduce PTSD symptoms in nurses.

## Data availability statement

The original contributions presented in the study are included in the article/supplementary material, further inquiries can be directed to the corresponding author.

## Ethics statement

The studies involving human participants were reviewed and approved by the Ethics Committee of the School of Nursing, Yangzhou University (No: YZUHL2021028). The patients/participants provided their written informed consent to participate in this study. Written informed consent was obtained from the individual(s) for the publication of any potentially identifiable images or data included in this article.

## Author contributions

MX: conception and design of the study, writing the original draft, analysis of data, and data curation. YY, HC, and MD: resources and acquisition of data. YL: conception and design of the study, writing the review, and supervision. HS and JiQ: analysis of data. TZ and JZ: conceptualization. JuQ, YB, SJ, YH, ZY, YF, and XG: data curation. All authors contributed to the article and approved the submitted version.

## Funding

This work was supported by the 2020 Medical Education Research Project of the Medical Education Branch of the Chinese Medical Association and Medical Education Professional Committee of the Chinese Society of Higher Education (Grant Number 20A0576), the Jiangsu Graduate Research and Innovation Program (Grant Number KYCX21_3298) and the Interdisciplinary Research Project of Chinese Language and Literature Special Zone of Yangzhou University (Grant Number yzuxk202019).

## Conflict of interest

The authors declare that the research was conducted in the absence of any commercial or financial relationships that could be construed as a potential conflict of interest.

## Publisher's note

All claims expressed in this article are solely those of the authors and do not necessarily represent those of their affiliated organizations, or those of the publisher, the editors and the reviewers. Any product that may be evaluated in this article, or claim that may be made by its manufacturer, is not guaranteed or endorsed by the publisher.
